# Developing Sustainable Chemistry with AI

**DOI:** 10.1021/acs.accounts.6c00552

**Published:** 2026-10-05

**Authors:** Jiyizhe Zhang, Polina Yaseneva, Alexei Lapkin

**Affiliations:** † Department of Chemical Engineering and Biotechnology, 2152 University of Cambridge, Cambridge CB3 0AS, United Kingdom; ‡ Department of Chemical Engineering, 5292 University of Manchester, Manchester M13 9PL, United Kingdom; § Department of Chemical Engineering, University College London, London WC1E 6BT, United Kingdom; ∥ Cambridge Centre for Advanced Research and Education in Singapore (CARES), 1 Create Way #05-05 Create Tower, Singapore 138602, Singapore; ⊥ Chemical Data Intelligence (CDI) Pte Ltd, 105 Cecil Street, The Octagon #22-25, Singapore 069534, Singapore

## Abstract

Today we are experiencing an unprecedentedly rapid transformation of science, driven by advances in computing and artificial intelligence (AI). At the same time, there are growing cumulative pressures of climate change impacts that not only affect economies but are creating significant societal challenges. The confluence of technology push and environmental–socioeconomic pressures is promoting the development of sustainable chemistry. The link between the transition to sustainability in chemistry and the digital transformation of R&D and manufacturing has been the long-standing focus in our research (Fantke, P.; et al. *Chem* **2021**, *7*, 2866 doi: 10.1016/j.chempr.2021.09.012). Sustainability is not a feature of a single chemical product or a process. It is incorrect to talk about “sustainable polyethylene”, as such narrow framing will miss many issues in the more holistic system that not only includes the cradle-to-grave life cycle emission impacts of polyethylene but also considers all aspects of equity, impact on biodiversity, resilience of supply chains, etc. Sustainable chemistry makes sense only as a complex system. An analysis of complex systems requires large amounts of data and the use of appropriate analytical tools, such as advanced machine learning techniques.

The chemical industry has been an early adopter of digital technologies, motivated by the inherent complexity of chemical systems and the need for efficiency to stay economically competitive. As early as the 1970s, cheminformatics tools were introduced to support structure–property analysis. In the 1980s, we saw the broad adoption of process modeling and simulation tools for design, scale-up, and optimization of chemical processes, paralleled with developments in the field of process system engineering and control. Today’s digital transition is much deeper; everything from scientific discovery (molecules, materials, and phenomena) to chemical process development, analytical methods and process analytical technologies, and plant operation is being reshaped. This deep transformation includes the development of a radically different approach to data in chemistry. Advances in self-driving laboratories, agentic AI workflows, and digital factories require access to structured, semantically rich data.

In this Account, we focus on three key areas of digitalization and AI technologies advancing chemistry and chemical process development toward net-zero targets. First, we discuss how data mining tools are used to analyze the existing value chains and identify opportunities to integrate net-zero feedstocks. When new molecules are introduced, data-driven route planning can be employed to design alternative synthetic pathways, which then need to be rigorously evaluated. Second, we focus on how to use AI to accelerate discoveries in chemistry, including the innovative design of catalysts, rational selection of solvents, and identification of optimal reaction conditions. By leveraging statistical modeling and data-driven optimization, these traditionally time-consuming tasks can now be performed much faster. Lastly, we discuss how to bring these innovations from the laboratory to manufacturing, which also requires advances in process technologies. We show that by leveraging AI agents, process modeling, and knowledge graphs, there is a potential to achieve automated process design and life cycle assessment, removing traditional choke points in scaling technologies and providing timely support for decision-making in early-stage development. In the end, we discuss the current challenges and future perspectives.

## Graphical abstract

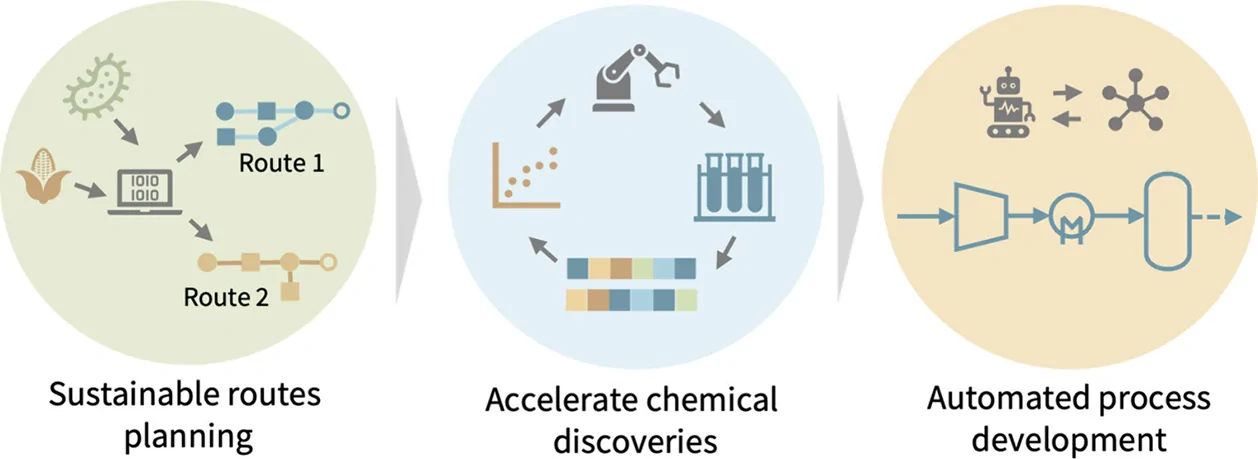

## Key References

-

Fantke, P. ; Cinquemani, C. ; Yaseneva, P. ; De Mello, J. ; Schwabe, H. ; Ebeling, B. ; Lapkin, A. A. Transition to Sustainable Chemistry through Digitalization. Chem 2021, 7, 2866–2882 10.1016/j.chempr.2021.09.012 .^1^ DOI: 10.1016/j.chempr.2021.09.012. *A perspective paper arguing for the critical role of digital infrastructure for development of sustainable chemical solutions.*
-

Weber, J. M. ; Guo, Z. ; Lapkin, A. A. Discovering Circular Process Solutions through Automated Reaction Network Optimization. ACS Eng. Au 2022, 2­(44), 333–349 10.1021/acsengineeringau.2c00002 .^2^ *Introduction of a novel automated framework that combines reaction network generation, kinetics, and optimization to systematically identify circular and resource-efficient chemical processes, reducing the manual design efforts and accelerating discovery of sustainable processes.*
-

Seidenberg, J. R. ; Khan, A. A. ; Lapkin, A. A. Boosting Autonomous Process Design and Intensification with Formalized Domain Knowledge. Comput. Chem. Eng. 2023, 169, 108097 10.1016/j.compchemeng.2022.108097 .^3^ *The developed hierarchical reinforcement learning agent system interacts with an ontological knowledge base to discover the optimal intensified process designs, demonstrating great potential of using knowledge representation to empower the performance of intelligent agents.*
-

Laub, J.-F. ; Zhang, J. ; Heyer, M. ; Lapkin, A. A. Automated Generation of Mechanistic Models for Chemical Process Digital Twins Using Reinforcement Learning Part II: Compartmentalization and Learning-Based Recalibration. Comput. Chem. Eng. 2026, 204, 109384 10.1016/j.compchemeng.2025.109384 .^4^ *Design a novel pipeline for automated generation of physical models for novel design of unit operations using colored graphs and reinforcement learning. Embed human knowledge into the workflow by defining graph grammar and reward shaping.*
-

Zhang, S. ; Zhang, J. ; Lapkin, A. A. A Knowledge Graph Framework for Digital Twins of Chemical Processes. Nature Chemical Engineering 2026, 3, 286–297 10.1038/s44286-026-00392-1 PMC1321215842211390 ^5^. *The fundamental approach to handling chemical process knowledge through a knowledge graph; we introduce and expand the necessary ontologies and show how this technology is supporting multiple applications in process development.*

## 1 Introduction

The chemical industry is fundamental to modern civilisation; while many tasks in the “service economy” depend largely on access to electricity, most industries depend on molecules and formulations of molecules and materials. We design molecules and materials that perform desired functions in a wide range of applications. We develop synthetic and purification procedures and combine them into manufacturing processes. Production, use, and final disposal of functional molecules and materials come at the cost of used resources and of potentially harmful emissions.

Focusing on climate change, it is estimated that the chemical sector accounts for approximately 6% of global CO_2_-equivalent emissions.^6^ Apart from direct emission of CO_2_ from chemical manufacturing, around one-third of total emissions come from processing fossil feedstocks and end-of-life product treatment.^7^ Tracing emissions and allocating them to individual stages within chemical value chains is a significant and urgent problem.^8^ Having a clear picture of the sources of emissions is critical to our ability to construct more-efficient value chains.^1^

Moving carbon from fossil stores to the atmosphere through the current linear chemical value chains and the generation of further CO_2_-eq emissions in processing and end-of-life are the two core challenges that can be addressed through a holistic re-engineering of the whole chemical value chains. For this, the whole-system approach is required, which considers (i) shifting from linear to circular supply chains (with biorenewable, captured CO_2_ and recycled net-zero feedstocks), (ii) designing novel synthetic procedures, (iii) linking chemical manufacturing with nonfossil energy generation and storage, and (iv) introducing innovations in process technologies, which would drive down the global metrics of resource use, emissions, and costs evaluated by cradle-to-grave life cycle assessment (LCA).^9^ When we add other aspects of sustainability to the challenge of “net-zero chemistry”, our design vector for molecules and routes becomes even more complex (see Figure 1).

**Figure 1:**
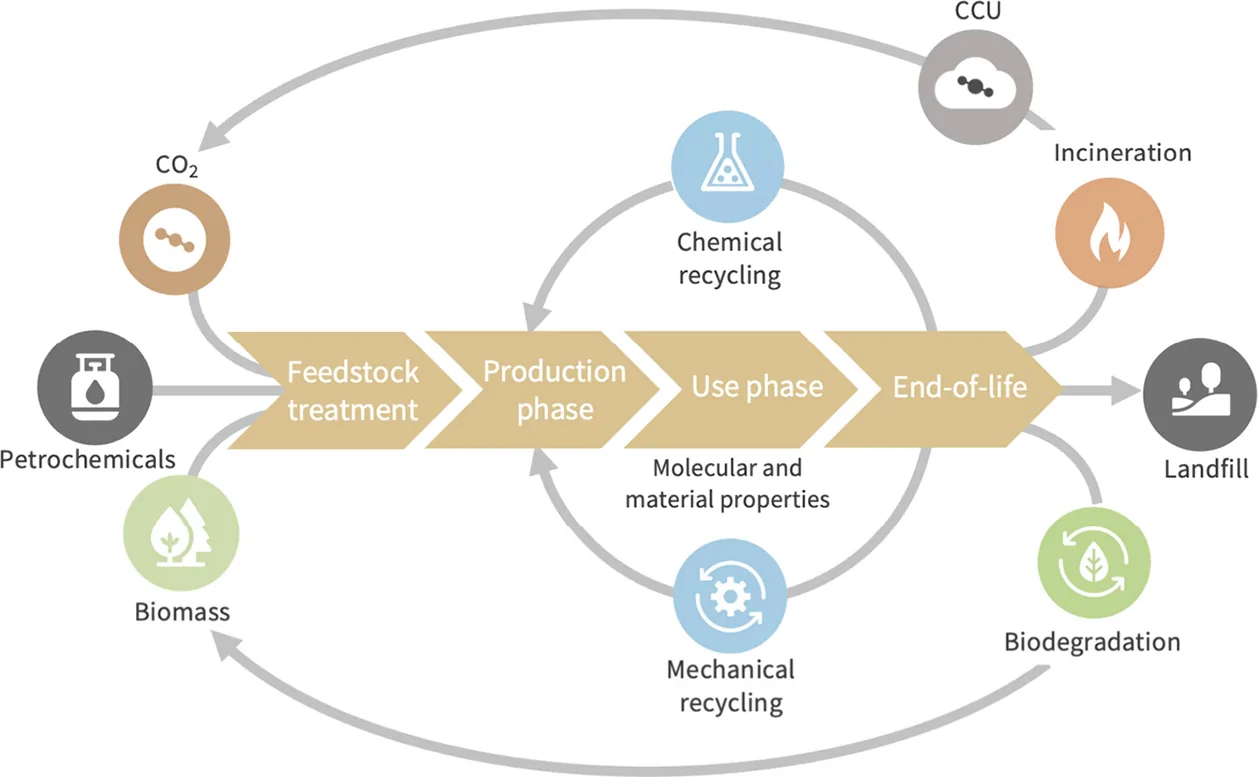
A schematic illustration of the emerging circular multifeedstocks paradigm of sustainable chemistry.

We argue that the system-level challenge of developing sustainable chemistry requires digitalization of chemistry knowledge and chemical manufacturing technologies, and the adoption of artificial intelligence (AI) analytical tools.^1^ The chemical industry has been one of the pioneers of digitalization and AI, specifically in the areas of cheminformatics for chemical synthesis and molecular property prediction,^10–12^ developing machine learning (ML) surrogates for process modeling and optimisation,^12^ and applying expert systems for process design.^13^

Increasing availability of data and computing power are beginning to translate into an increased rate of scientific discovery; application of digital tools and AI to solving problems in science and engineering is becoming the new normal,^14^ as highlighted by the Nobel Prize of 2024. It is a good time to consider how we could leverage these new capabilities to help us accelerate innovation in chemistry and chemical manufacturing for the transition to a sustainable chemical industry.

In this Account, we explore this theme. We provide an overview of how AI can support chemistry research and process development toward a sustainable future by focusing on three aspects: design of sustainable value chains, acceleration of chemical discoveries, and development of chemical processes. Challenges and future directions are discussed at the end.

## 2 Sustainable Chemical Value Chains

Today’s chemical value chains begin primarily from fossil resources (oil, coal and natural gas) as the main sources of carbon, heteroatoms, and process energy. In the linear economy with many products having short usable lifetimes, this leads to chemistry serving as a pump of fossil carbon into the atmosphere, contributing to climate change. Long-lived chemicals are contributing to long-term pollution (plastics and “forever chemicals” in the environment). As we strive to move chemistry to the delivery of societally useful functions without contribution to climate change (“net-zero chemistry”) and other environmental problems through the adoption of new frameworks such as Safe and Sustainable by Design,^15^ we are considering the nature of the feedstocks used, efficiencies of the procedures, and nature of energy and waste streams simultaneously.

A shift away from fossil feedstocks would require the adoption of a diverse set of feedstocks, including fresh renewable biomass, biowaste, captured and upgraded CO_2_, and recycled carbon/heteroatoms from end-of-life streams^16,17^ as alternative sources of elements. The new set of raw materials leads to either the same (drop-in) or a new set of intermediates.^6^ We found it productive to discuss intermediates in the context of their role within reaction networks as “effective connectors”, thus defining them as “hub” or “strategic” molecules.^18^

The “drop-in” strategy is when net-zero feedstocks are converted to produce a “hub” molecule that is a part of an existing value chain. This approach benefits from the use of existing manufacturing infrastructure. An example is the development of direct chemical transformations of lignocellulosic materials to produce platform molecules such as 5-hydroxymethylfurfural (HMF) or developing a route from terpenes to terephthalic acid, leading to a biobased monomer;^19^ these drop-ins can link seamlessly into the existing downstream processes.

The second approach is more disruptive and product-oriented, focusing on developing entirely new products and pathways, such as producing Cyrene from cellulose to replace the toxic solvent *N*-methyl-2-pyrrolidone.^20^ This case is particularly interesting as Cyrene may not necessarily be used as a solvent but as a new starting structure in the synthesis of more complex functional molecules; in other words, it may become a new “hub” molecule. This also applies to novel materials that reduce the use of fossil resources and offer new functionalities, such as the emerging nanocrystalline cellulose-based products.^21^ For both approaches, new reaction pathways need to be identified or developed to link with new feedstocks. We explored data mining and algorithmic methods to enable an automated search for sustainable routes.

### 2.1 Reaction Data Mining and Route Planning

Any given value chain of molecules is a fragment of a much larger network of reactions. This earlier concept was revived by Grzybowski and co-workers,^22^ demonstrating that a global view on reaction networks provides insights for reactivity analysis and for guiding synthesis planning. Within such networks there exist highly connected “hubs” which appear in most synthetic routes.^23^ Here we are connecting the mathematical term “hub” from scale-free networks with practical meaning in chemical synthesis. We first identified hub (strategic) molecules by defining their mathematical features and identifying them within a large network of molecules as outliers.^18^ We further hypothesized that the transition of chemical value chains away from fossil carbon sources will lead to the emergence of new strategic molecules,^24^ the concept we are currently investigating.

Having access to a majority of the currently known chemical reactions, for example, through exploring Reaxys as a source of data for reaction networks,^25^ allows us to explore synthesis planning with the primary focus on sustainability: using biorenewables as starting molecules,^26–28^ generating multiple metrics to enable multicriteria decision making in ranking promising routes,^29^ and optimizing mass flow based on exergy through a reaction network, for example, by using Petri nets.^30^ An automated pipeline has been developed to integrate data mining, network analysis, and pathway planning and assessment,^2^ which significantly reduces the effort of evaluating promising routes and reduces the barrier of adopting these technologies in the early stage of process development (Figure 2).

**Figure 2:**
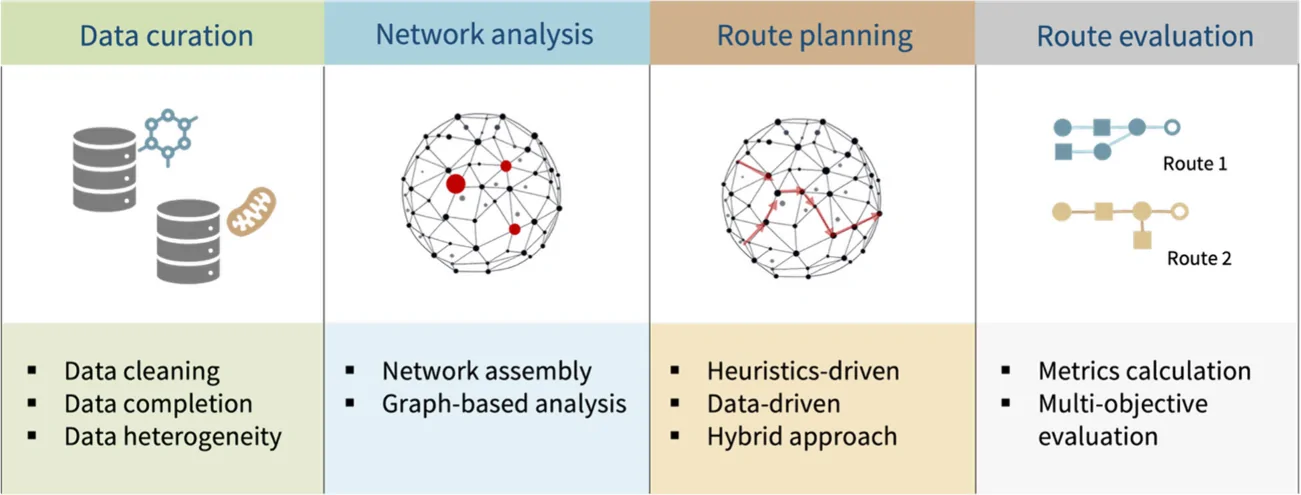
A schematic illustration of a workflow of reaction data mining and synthetic routes planning.

### 2.2 Introduction of Net-Zero Feedstocks

There is a particular challenge in designing sustainable routes toward molecules containing heteroatoms. Some biorenewable feedstocks (e.g., lignocellulose) contain carbon-, hydrogen-, and oxygen-rich molecules, whereas nitrogen-containing building blocks are found in very different renewable feedstocks (chitin or proteins) or through potential net-zero ammonia routes. As a result, the synthesis planning may require access to multiple feedstocks. In a recent study, we used a deep data mining approach based on similarity-based heuristics and in-depth molecular structure analysis to identify promising synthetic pathways for the production of pyrrole-2-carboxylic acid using both cellulose and chitin as raw materials.^26^ Several promising synthetic routes were identified and subsequently verified experimentally. In the broader literature, data-driven approaches complement graph methods and are being actively developed for route design; a recent review of this topic can be found elsewhere.^31^

Advanced route planning tools allow exploration of the impact of novel synthetic methods on overall synthesis planning. Developments of new synthetic methods such as electro-, photo-, and biocatalytic reactions are enabling novel bond disconnections and introducing new options for route design. In a recent study, we have demonstrated the design of synthesis routes that include both chemical and biochemical reactions.^32^ For this, we built a database of feasible bio- and chemical transformations and used reinforcement learning for optimizing synthetic paths. Such an approach allows one to bring novel synthetic methods into synthesis planning.

## 3 Acceleration of Discoveries in Chemistry and Chemical Processes

Innovation in chemistry has been a protracted and costly process, with discoveries often made through serendipity, while development timelines often stretch over decades.^33^ Today, our aspiration is to significantly reduce the chemical development timeframes through a combination of several key technologies: (i) AI tools for molecular/materials design, (ii) automation and AI enabling faster data generation in chemical reaction development and in process conditions exploration/optimization, and (iii) adoption of new synthetic methods. We are particularly interested in technologies that allow significant acceleration of the development timelines for chemical reactions: forward and inverse design, automated optimization, and incorporating prior knowledge through chemical descriptors.

### 3.1 Forward and Inverse Design

Forward design in the context of chemical reactions takes the form of trial-and-error testing of molecules, reaction conditions, catalysts, and materials that allow us to obtain the target result. For example, the identification of an optimal catalyst for a specific reaction is typically a screening of available options. This approach to computational catalyst design is through forward enumeration—exploring the potentially feasible catalytic structures and then screening for reactivity and selectivity in the desired transformations. This can be done for both homogeneous and heterogeneous catalytic reactions.^34–36^ Here we can make use of the growing number of data sets of material structures and Density Functional Theory (DFT)-computed energies.^37,38^ The accuracy of predictions based on DFT inputs and the size of the data set are conflicting demands, as accurate calculations of equilibrium geometries and of activation energies by an appropriate level of DFT theory are highly expensive calculations. Hence, we note that currently only limited types of catalysts are considered in each individual study. A rapidly developing field of machine learned force fields is promising in attaining the required level of accuracy of energy prediction to be able to substitute expensive DFT calculations with the much faster force field methods, which will revolutionize the field of catalyst design.^39^

Inverse design aims to identify the solution directly from the set of target properties (e.g., identifying the optimal catalyst structure directly from the specification of the target reaction). For the inverse design of catalysts, we may imagine a workflow when we start by identifying plausible energy diagrams, or reaction coordinates, that enable a selective transformation from starting molecules to the desired product(s). Identification of potential feasible energy diagrams can be formulated as an optimization problem that has multiple solutions. Once we have a set of possible potential energy diagrams, we would want to identify what catalysts may enable such reaction coordinates. However, the next step, the inverse materials design challenge, is much harder. For the inverse design methodology to be developed, we need to link fundamental theoretical approaches allowing forward predictions with machine learning models built on experimental or computational observations. As an example, a library of water–gas shift catalysts and their experimental studies was assembled; a neural network was trained on the data set which then was used to design a new heterogeneous catalyst.^40^ The use of statistical machine learning models is a very fruitful direction for materials and formulations design, areas where large sets of high-quality experimental data may exist. Such models are limited in their predictive capacity since the models usually do not allow us to predict materials that are not present in the data set. However, some extrapolation may be feasible if catalysts/materials are parametrized and machine learning models are trained on “descriptors” rather than the instances of specific materials.^41^ A recent review describes state-of-the-art and potential directions for the future development of computational methods of catalysts design.^42^

### 3.2 Reaction Development with ML and Self-Driving Laboratories

Reaction systems involve combinations of catalysts, solvents, and operating conditions (e.g., temperature, pressure, and concentration), creating a high-dimensional space where identifying optimal conditions for later scale-up can be challenging. This is effectively addressed by multidimensional optimization in closed-loop experiments (Figure 3(a)). Two key techniques are combined: a surrogate function that captures the complexity of a multivariate problem, and an efficient sampling method that reduces the number of observations (experiments), and hence the experimental effort required to identify optimal conditions. When an efficient sampling algorithm is combined with an automated experimental system, we obtain a closed-loop or a “self-driving” experiment.^43,44^

**Figure 3:**
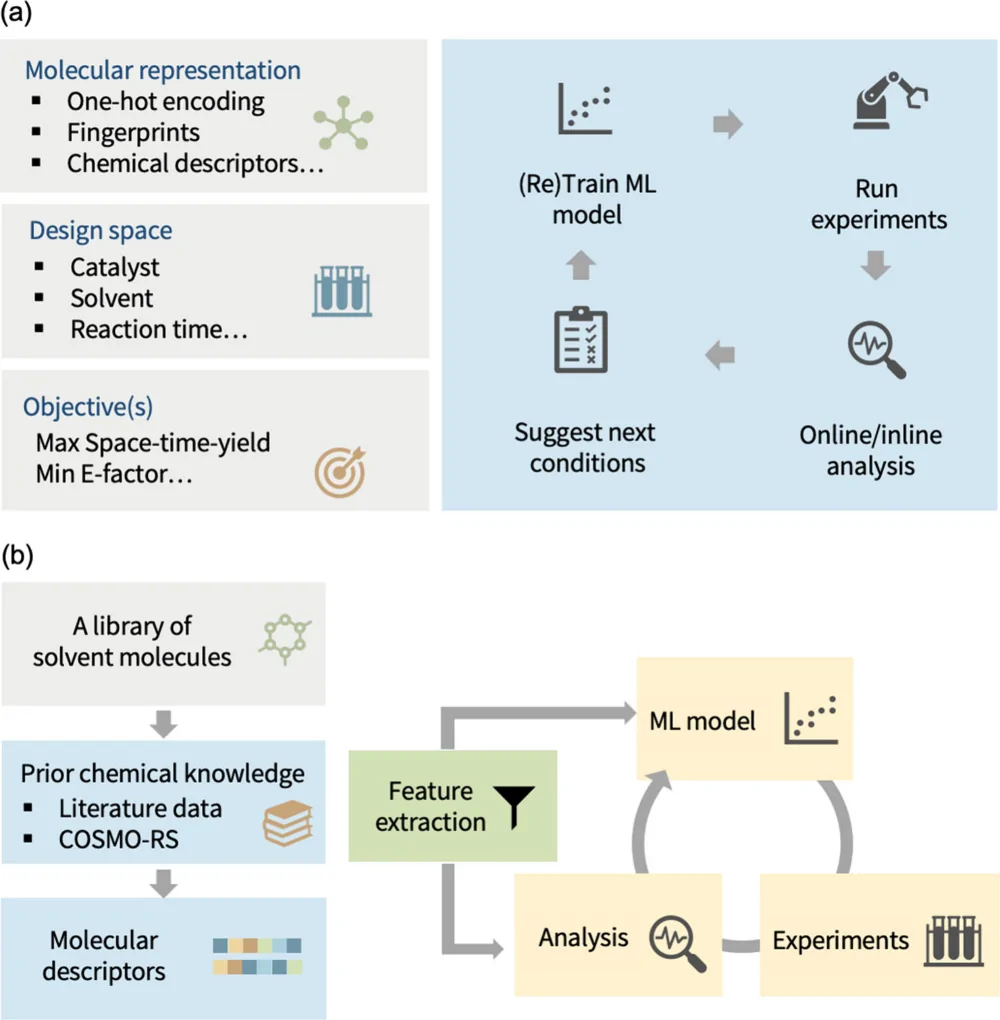
Applications of AI for the development of chemical transformations. (a) Closed-loop reaction optimization assisted by Bayesian optimization and (b) use of chemical descriptors to assist solvent selection.

The algorithmic side of “self-driving” laboratories has seen rapid and widespread development. See Table 1 for an overview of available packages. Initially, we worked with algorithms in which multiple objectives (e.g., reaction yield and cost) were scalarized to a single objective.^45^ Later, we moved to multiobjective Bayesian optimization algorithms.^46^ Notably, these algorithms can be used in manual^47^ and semiautomated^48^ workflows as well as within fully automated “self-driving” laboratories. A comprehensive review of the development of self-driving laboratories can be found elsewhere.^49^

**Table 1:** A Noncomprehensive List of Open-Source Python Packages for Reaction Optimization^a^

Package	Description	Link
Gryffin^50^	SOBO for both continuous and categorical variables	https://github.com/aspuru-guzik-group/gryffin
NE Torch^51^	MOBO for continuous variables	https://github.com/VlachosGroup/nextorch
Summit^52^	SOBO and MOBO for both continuous and categorical variables	https://github.com/sustainable-processes/summit
BayBe^53^	SOBO and MOBO for both continuous and categorical variables	https://github.com/emdgroup/baybe
BoFire^54^	SOBO and MOBO for both continuous and categorical variables	https://github.com/experimental-design/bofire

a SOBO, single-objective Bayesian optimization; MOBO, multi-objective Bayesian optimization.

### 3.3 Chemical Knowledge Representation via Descriptors

A specific subproblem in the field of reaction development is the challenge of chemical knowledge representation.^55^ Here, chemical descriptors are a way of enriching the data available for training machine learning models by encoding knowledge such as physical chemistry, molecular/material structures, interactions of different length scales in specific *descriptors*. Because the databases of known chemical facts are very small and rarely explore facts systematically, it is challenging to rely on ML to learn chemistry from observations. The addition of chemical descriptors in many cases allows an improvement of the accuracy of ML predictive models.

A particular success story with the use of molecular descriptors is the prediction of the role of solvents in reactions. Solvents are ubiquitous in chemical transformations, providing the environment in which chemical reactions occur as well as serving as useful process functions such as heat transfer. Solvents significantly influence reaction rates and selectivity, either enhancing or reducing yields.^56^ The selection of a good solvent is often guided by experts’ experience or past literature. A computer-aided approach based on DFT calculations leverages quantum chemistry to predict solvent properties,^56^ which allows for screening a set of solvent candidates. However, these methods are computationally expensive.

Amar and Zhang developed automated workflows for the rational selection of solvents for catalytic reaction systems.^57,58^ Machine learning surrogates were trained based on molecular descriptors from the literature as well as descriptors based on physical knowledge from COSMO-RS simulations. These surrogates were then used in Bayesian optimization to successfully identify the best solvents to meet two targets simultaneously, namely, high conversion and high diastereomeric excess (Figure 3(b)), or the more efficient Mitsunobu reaction conditions.

## 4 From Laboratory to Manufacturing

For many chemical processes, the transition from laboratory discoveries to manufacturing is very challenging. This is true for scaling manufacturing of small molecules, biologics, nanomaterials, or any processes involving multiple phases. To successfully scale a multiphase process, it is critical to understand the variation in process *context* and to track the changes that must be implemented to attain the desired product quality and process key performance indicators (KPIs). The concept of context is becoming particularly important in both chemistry and AI. In chemistry, context is critical in developing correct first-principles models and for scaling.^59^ In AI, context has different meanings, from a collection of facts to the assembly of text chunks exposed to a language model. The transition to digital R&D and digital manufacturing is promising to make the challenge of scaling processes more tractable, especially with the advances in agentic AI frameworks and digital twin technology (Figure 4).

**Figure 4:**
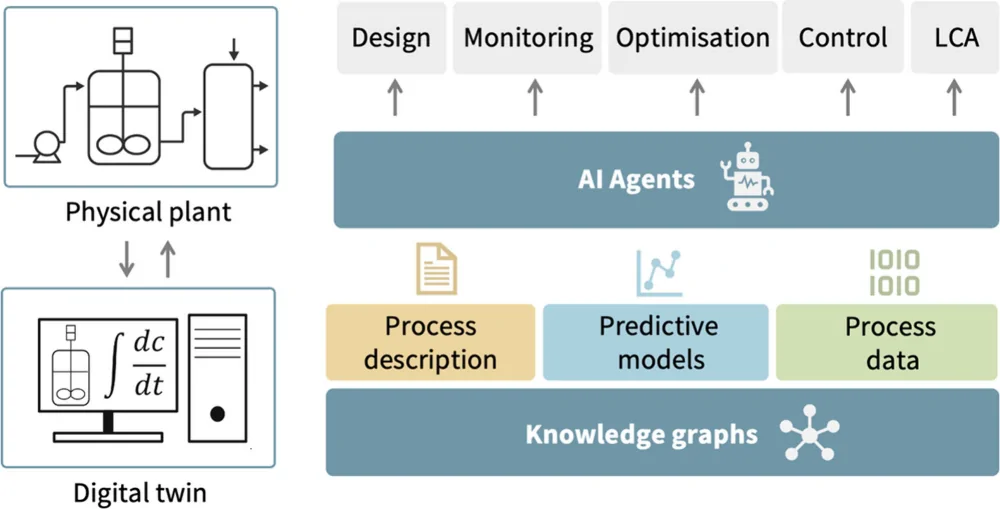
An illustration of the concept of the digital twin and AI agentic system.

### 4.1 *In Silico* and Automated Process Design

The combination of predictive models and knowledge graphs is highly promising for creating “digital twins” of chemical processes and enabling *in silico* process design, which can also be extended to automated process design and full plant operation in the agentic framework.^60^ For the case of finding an optimal flowsheet composed of intensified process technologies, a generalized methodology was proposed by Sundmacher and Freund,^61^ which is based on optimizing a path through a thermodynamic state space from starting conditions to the desired products, and can be framed as a flux optimization problem.^62^ Identifying such an optimal path and assembly of processing options can be addressed through hierarchical reinforcement learning (RL), one of the AI methods which aims to find a successful strategy of winning in a game given a set of rules.^63^ Recently, we developed a hierarchical RL methodology for automated process design.^3,64^ To provide information to RL on feasible steps to take, we give it access to a knowledge store in the form of a *knowledge graph* of fundamental process phenomena.

A knowledge graph is an assembly of facts and their relationships. In our case, we store knowledge of possible molecular and energy fluxes relevant to specific chemical tasks, such as synthesis, separation, etc. This knowledge base is used to assemble potential designs within an RL environment. The environment provides data on the efficiency of the created design. The RL process has access to information about all past trials—model evaluations—and guides design toward the most rewarding strategy. Ultimately, this approach leads to a ranked set of potential flowsheet designs (Figure 5(a)).

**Figure 5:**
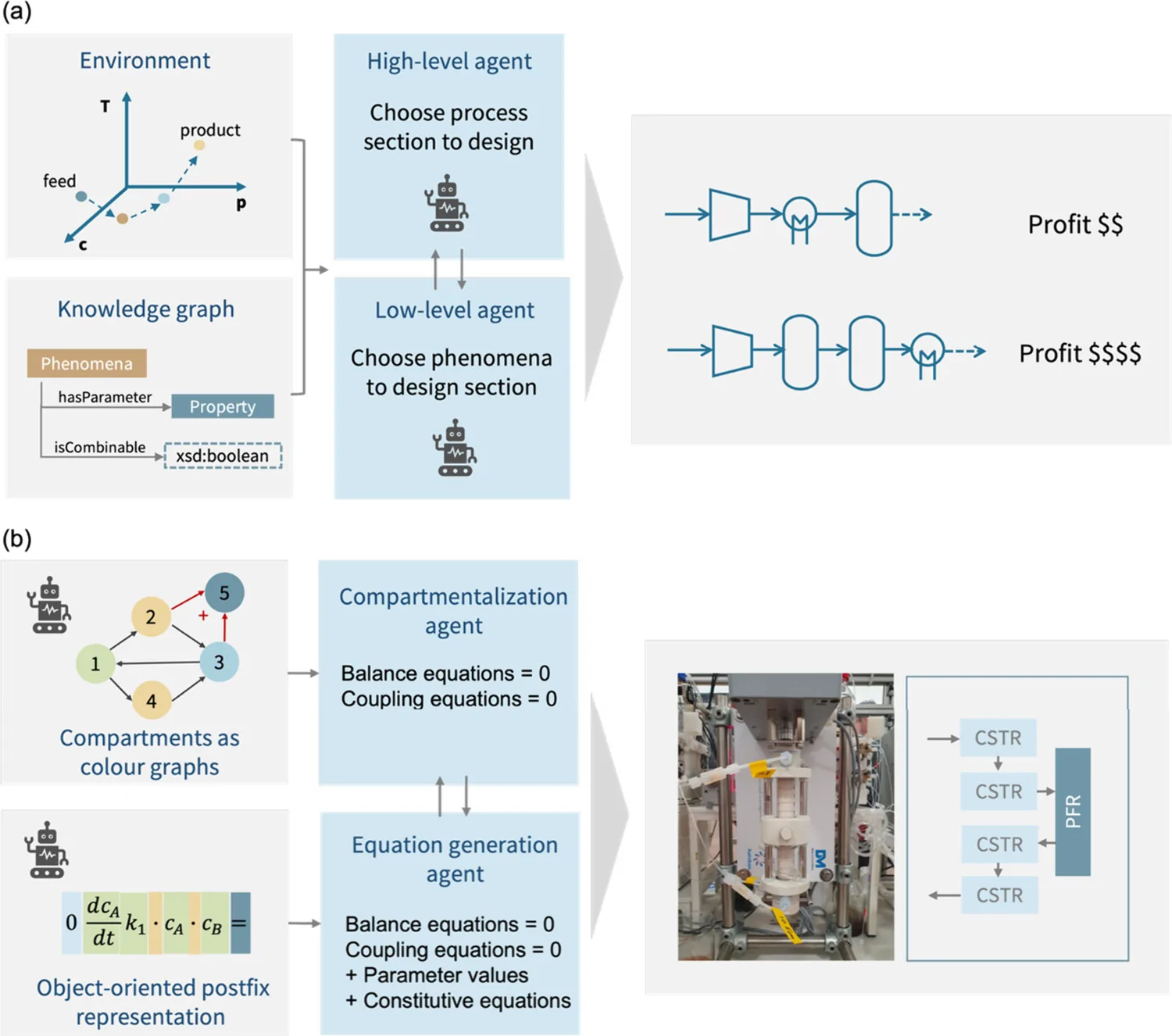
Reinforcement learning agents for (a) automated process design and (b) modeling of novel reactors.

A similar RL methodology can be adopted to automatically assemble first-principles models of reactors or devices, such as new mixing designs, compartmental membrane reactors, and multiphase catalytic systems. The modeling of such systems can similarly be formulated as a hierarchical RL problem (Figure 5(b)). We have recently proposed an overall pipeline for automated model generation and demonstrated its performance on creating a hydrodynamic model of a Taylor-Couette flow reactor.^4,65^ The pipeline starts with a library of process phenomena and produces executable process models. We explored different ways of formalizing human knowledge into the RL agentic workflow by defining graph grammar or reward shaping. We also observed that the modeling ability learned by the agent is transferable from one scenario to another, showing the potential of reuse and generalization of the strategy.

### 4.2 Automated Life Cycle Assessment (LCA)

In developing novel technologies, a key focus is on improvements in the environmental impacts of novel technologies over the industry benchmarks in the delivery of similar functions. The evaluation of environmental impacts is best done through life cycle assessment (LCA), a comprehensive and systematic evaluation of environmental impacts over the full cradle-to-grave life cycle of a product or process against a defined function. While we are now able to automatically construct individual models and even optimal flowsheets, the next challenge is to be able to perform automated LCA of complete process sequences to support process design and decision making.

Our early example of integrating LCA with process models for automated process optimization was shown in a study by Helmdach and Yaseneva et al.^66^ Here a two-step chemical conversion–separation sequence of terpenes into useful intermediates was optimized by a multiobjective Bayesian optimization algorithm which had access to a rigorous process model to estimate economic costs and LCA software for the evaluation of environmental impacts.

A challenge to fully automate LCA is the generation of reliable life-cycle inventory data, which is limited by the availability of high-quality, publicly accessible data. One potential solution is to use rigorous process simulation^67^ to estimate missing inventory data, which could be further supported by automated process design and model-generation workflows. Another approach demonstrated in the literature is to use a decision tree model to estimate the missing data in life-cycle inventories.^68^ Such a model was trained on gate-to-gate inventory data from 409 organic chemical processes. This method is based on stoichiometric reaction data, which simplifies the calculation of the required amounts of raw materials. The authors demonstrate improved accuracy of predictions for some impacts but note that the uncertainty of the predictions is still very high. Another option is to train statistical ML models based on existing databases to directly link molecular structures with their life-cycle environmental impacts.^69,70^ This approach generally leads to very large uncertainties due to very small available training data sets and the inability of capture process variability by models trained on so little data.

## 5 Challenges and Outlook

Rapid evolution of computing and AI is reshaping the way we design chemistry and chemical processes and will affect manufacturing. Several challenges and future development directions are discussed here.

### 5.1 Data

Data remain a major bottleneck in chemistry R&D. This concerns the availability of historical data and the generation of new high-quality data.

For the historical data, many challenges are well-publicized: data may exist in diverse formats, have arbitrary coverage (e.g., unequal exploration of reactivity patterns of molecules with different substitution patterns or a lack of studies of the effects of solvents in reactions), be poorly labeled or mislabeled (when a catalyst appears as a solvent in a record, etc.), or exist only behind paywalls or in-house company repositories. The issue of data quality in historical records is multifaceted. Apart from data gaps, there exists the issue of lack of standardization in how some data are collected or, as a minimum, a lack of metadata on how experiments were conducted, which would allow some normalization between data collected by different groups. There is an issue of data quality as a function of instrumentation used. Thus, early data on electronic microscopy may be only partially useful, as newer instruments provide significantly better resolution and may also help to reduce operator dependency in collecting high-quality images. Because of problems with access and the quality of historical data, many groups favor the generation of new data.

Advances and broad adoption of laboratory automation, high-throughput experimentation, and process analytical technologies (PAT) offer opportunities to generate data more efficiently. The combination of laboratory automation with intelligent sampling further improves the quality of collected data for specific applications. Advanced computational techniques, such as transfer learning, reduce the need for new data generation.^71^ In some cases, multidimensional data are needed, such as both temporal and spatial data to reveal details of transport phenomena in multiphase processes. Advanced sensing techniques such as hyperspectral imaging could potentially be useful to efficiently generate experimental multidimensional data sets.^72,73^

Lastly, while this Review primarily focuses on chemical reactions, both upstream and downstream process steps, such as the generation of renewable feedstocks or product separation and chemical products’ end-of-life conversion, are as important for the overall process design, and data efforts should extend to cover these fields as well.

### 5.2 Models

As famously noted, “all models are wrong, but some are useful”; models are never perfect. This goes for both first-principles and data-driven models. First-principles models reflect our understanding of physical reality. They are developed through a reductionist approach, generating predictions that allow us to study individual, or few, phenomena. Data-driven models and research methods allow us to reveal interactions in multidimensional spaces. They can enhance and sometimes replace the reductionist methodologies in the study of difficult problems, often promising a significant benefit in terms of lower experimental budgets, for example, through the use of active learning. A combination of first-principles models with ML methods, either in the form of physics informed neural networks or by using physical models as priors in Bayesian methods, allows us to solve inverse problems and provide interpretability to ML. New machine learning methods are also actively being developed to enhance the process of discovery of the underlying first-principles models from data, for example, through symbolic regression.^74^

### 5.3 Infrastructure

The increasing adoption of AI in research workflows requires better infrastructure for storing and using research data. This requires commitment to standardized data management aligned with FAIR (findable, accessible, interoperable, reusable) principles. Good practices of data management in structural biology enabled breakthroughs such as AlphaFold.^75^ Recent efforts in chemistry, such as the Open Reaction Database^76^ and materials ontologies,^77^ are actively advancing standardization for chemistry data. This will need to extend to process chemistry and chemical process design. Standardization does not necessarily mean one standard. Several standards may coexist, as long as their schemas are published. Here, the research community is rediscovering the results of computer science from the 1980 to 1990s. For example, the issue of interoperability of ontologies has been studied well before ontologies become an important topic.^78^ For chemistry data, recording the method of data generation and creating semantically enriched data “packets”, such as RO Crates,^79^ seem to be reasonably straightforward to implement. Data could be retrieved from RO Crates and prepared in specific data products to match the most suitable data format to the model being trained. Standardization is also critical for communication between hardware (e.g., between laboratory automation devices^80^) and software (e.g., interoperability between modeling platforms,^81^ between AI agents^82^). Researchers are encouraged to adopt and expand existing standards where possible.

### 5.4 Training Power and Investments

While digitalization and AI offer exciting opportunities for chemistry and engineering, it should be noted that adopting AI itself should be “sustainable” as well.^83^ Training large AI models demands substantial energy, often from nonrenewable sources. For example, training a 175-billion-parameter model can consume 1,287 million kWh—enough to power a town of 4,000 households for a day.^84^ Principles of smart computing—using appropriate tools for the problems at hand—should be taught, popularized, and encouraged, alongside other principles of responsible research and innovation frameworks. At the same time, we note that established research institutions are poorly prepared for fully digital R&D. From buildings designed for manual laboratories to ineffective IT management, outdated data centers and computational resources, and very slow timelines for funding decisions, all require radical rethinking in the very near time. Recent public release of a substantial cache of research tools by Google (Gemini for Science) and Anthropic (Claude Science) is indicative of another trend in science. Large corporations have built an infrastructure for conducting research using AI agents and are banking on selling access to their LLMs or agentic environments. Technology companies will be making it as easy as possible to “do research” using such frameworks, which will have several implications.

On a purely technical/commercial side, there is a risk of being locked into a specific provider of “science services”. “Own your AI” and “own your knowledge graph” are repeated warnings in technology blogs. On the positive site, digital infrastructure for science should lead to lowering the barrier to entry and reaching toward a much larger talent pull, likely significantly disrupting the status quo of few “power houses” of science. AI-assisted education is also developing very rapidly and should further support the development of a larger talent base. There is emerging evidence of enhancement in the rate of hypothesis evaluation and generation in the laboratories that actively use digital tools (AI, automation, and data integration).

On the risk side, we are entering a period of proliferation of “junk science”, much the same as we observe with AI-generated content in other fields. The sheer volume of mundane research outputs will require the development of new methods of scoring and filtering. Benchmarking of outputs and proof of ownership of data and ideas become critical to sieve out true talent from routine and to help promote genuine innovation. Finally, science may not be immune to the risk of over-reliance on AI for routine technical tasks, which are nevertheless required for skills training.
